# Mâmawihitowin (bringing the camps together): Perinatal healthcare provider and staff participation in an Indigenous-led experiential intervention for enhancing culturally informed care—a mixed methods study

**DOI:** 10.1186/s12939-022-01764-8

**Published:** 2022-11-16

**Authors:** Grant Bruno, Rhonda Catherine Bell, Brenda Parlee, Patrick Lightning, Ida Bull, Bruce Cutknife, Richard Thomas Oster

**Affiliations:** 1 Department of Pediatrics, University of Alberta, Edmonton Clinic Health Academy, 11405-87 Avenue NW, T6G 1C9 Edmonton, Alberta Canada; 2 Samson Cree Nation, T0C 1N0 Maskwacîs, Alberta Canada; 3 Department of Agricultural, Food & Nutritional Science, University of Alberta, 4-126B Li Ka Shing Centre for Research, 11203-87 Avenue NW, T6G 2H5 Edmonton, Alberta Canada; 4 Department of Resource Economics and Environmental Sociology, University of Alberta, 507 General Services Building, 9007-116 Street NW, T6G 2H1 Edmonton, Alberta Canada; 5 Ermineskin Cree Nation, T0C 1N0 Maskwacîs, Alberta Canada; 6 Faculty of Medicine & Dentistry, University of Alberta, 2-109A Edmonton Clinic Health Academy, 11405-87 Avenue NW, T6G 1C9 Edmonton, Alberta Canada; 7 Indigenous Wellness Core, Alberta Health Services, 306 Anderson Hall, 10959-102 Street, T5H 2V1 Edmonton, Alberta Canada; 8 Department of Community Health Services , Universtiy of Calgary , 3D10, 3280 Hospital Drive NW, T2N 4Z6 Calgary, Canada

**Keywords:** Indigenous health, First Nations, maternal health, Cultural competence, Perinatal care, Community-based participatory research, Qualitative research, Surveys and questionnaires

## Abstract

**Background:**

In partnership with a Nehiyawak (Plains Cree) community of Maskwacîs,central Alberta (Canada), we implemented an Indigenous-led intervention to provide experiential learning opportunities for perinatal health care providers (HCPs) and staff. Our objective was to capture the impact of participating in cultural safety learning opportunities on perceived self-awareness for HCPs and staff to provide enhanced culturally informed care.

**Methods:**

Perinatal HCPs and staff who work regularly with Indigenous women from our partnering community took part in a series of experiential learning activities designed by a Community Advisory Committee. We used an explanatory sequential mixed methods approach informed by community-based participatory research. We compared Cultural Intelligence Scale (CQS) and Maskwacîs-Specific Cultural Scale (MSCS) scores pre- and post-intervention using non-parametrical statistical analysis (Wilcoxon signed rank test). Post-intervention, we conducted a qualitative description study using semi-structured interviews. Qualitative data was analyzed using thematic analysis.

**Results:**

A total of 17 participants completed pre- and post-intervention questionnaires. Responses indicated a shift in perceived cultural and community knowledge and comfort levels, with positive gains in overall mean scores for both the CQS (p = 0.01) and MSCS (p = 0.01). Nine participants completed qualitative interviews. Overall, participants felt better equipped to provide more culturally informed care to their patients post-intervention.

**Conclusion:**

An Indigenous-led experiential learning intervention was effective in enhancing overall perceived cultural awareness and preparedness to provide culturally informed care for perinatal HCPs and staff. This study provides evidence for fostering relationships between Indigenous communities and health systems toward enhanced perinatal care.

## Background

Due to colonization maternal and infant health outcomes for Indigenous Peoples in Canada (First Nations, Métis, and Inuit) are generally poorer in comparison to non-Indigenous Canadians [1]. A lack of cultural awareness among health care providers (HCPs) and staff within the clinical setting is routinely cited as a major negative influence on the effectiveness of care for Indigenous patients [2, 3]. As such, racism, negative stereotyping of Indigenous women, and biases against their care is a pervasive problem within health care, as well as other aspects of social and political relations in Canada [4, 5]. Addressing this problem requires a shift in the institutions of care [6]. The Truth and Reconciliation Commission of Canada Call to Action 23.iii specifically states: “We call upon all levels of government to: provide cultural competency training for all health-care professionals” [7]. Building on a long-established community-based participatory research (CBPR) partnership with a Nehiyawak (Plains Cree) community in central Alberta, [8–10] we developed and implemented an Indigenous-led and culturally based intervention to provide experiential learning opportunities for perinatal HCPs and staff serving Indigenous women from the community. This study was an extension of the ENRICH Research Program, which aimed to understand the perceptions and experiences of diverse groups of pregnant and postpartum women with respect to environmental and social means to support healthy pregnancies, as well as identifying needs and opportunities to promote optimal maternal health.

In the Nehiyawin language, Mâmawihitowin loosely translates as “bringing the camps together for a common goal.” This concept was captured in the process of our collaborative research and the study objective: to capture the impact of participating in cultural safety learning opportunities on perceived self-awareness to provide culturally informed care. Experiential learning can be described as the learner having a direct experience on which they will reflect on, and from which they will synthesize new perspectives and knowledge. They then will apply and test this learning in future situations [11]. This approach to learning is widely used in Nehiyaw culture.

## Methods

### Research partnership, ethics, and oversight

This study was conducted in partnership between Maskwacîs (collectively, Samson, Montana, Louis Bull, and Ermineskin Cree Nations; total population ~ 16,000), Maskwacîs Health Services (MHS), the Wetaskiwin Primary Care Network (PCN), and University of Alberta researchers. Since 2013, our CBPR partnership has worked to understand the unique needs of women and families within the community and to improve women’s perinatal health and family well-being using strengths-based, community-led strategies. A Community Advisory Committee (CAC) guided this project, which included a core group of community Elders (authors PL, IB, and BC), a community member/graduate student (GB), and an academic researcher (RTO). The CAC was selected based on established kinship and relationships, and met regularly to review and decide on all aspects of the research.

### Ethics 

Ethical approval was provided by the University of Alberta Research Ethics Board (Pro00073909), which adheres to Chap. 9 of the *Tri-Council Policy Statement 2*, “Research Involving the First Nations, Inuit and Métis Peoples of Canada,” [12] and we obtained community approval from key health leadership, Elders, and community members. A collaboratively developed research agreement was ratified, outlining the purpose, approach, roles and responsibilities, methods, dissemination, ethical issues, and data ownership, including the principles of OCAP**®** (Ownership, Control, Access, and Possession) [13]. All data related to this study is co-owned by the community and researchers. As per the request of community partners and Elders, all data is stored securely at the University of Alberta and is made available to the community if/when needed. Intellectual property, such as collected data, traditional knowledge, and the sharing of such knowledge in research outputs, is guided and decided upon by the CAC. All research participants provided written informed consent.

### Study design

We used an explanatory sequential mixed methods design, [14] with CBPR as the overarching framework. Detailed conversations with the CAC led to the selection of a mixed methods design that included initial quantitative data collection (pre- and post-intervention) that informed subsequent qualitative data collection (post-intervention). The two types of data were integrated during the data interpretation phase. CBPR recognizes that community members are best positioned to understand their own context and hold knowledge crucial to research that benefits their community [15].

### Intervention design

Meetings with community members and the CAC helped identify issues that were community-specific and pressing, such as addressing gaps in perinatal care, racism in health care, and incorporating ceremony and spirituality into care. Our team and the CAC understood early on that cultural awareness training designed from western models and theories would not effectively address the wants or needs of Maskwacîs communities. Guided by established CBPR principles [16–18] and the CAC, we used a decolonizing approach to conceptualize the intervention. There is no unifying theory to decolonize research, rather it is an intuitive approach and prioritizes the situated knowledges of Indigenous individuals and communities [19–21]. For our study, these knowledges included traditional knowledge from Elders in the community as well as Nehiyawak language, ideas, and concepts. Understanding Mâmawihitowin helped create opportunities to embed local knowledge and culture into the intervention design (such as incorporating ceremony into all aspects of the research).

**Fig. 1:**
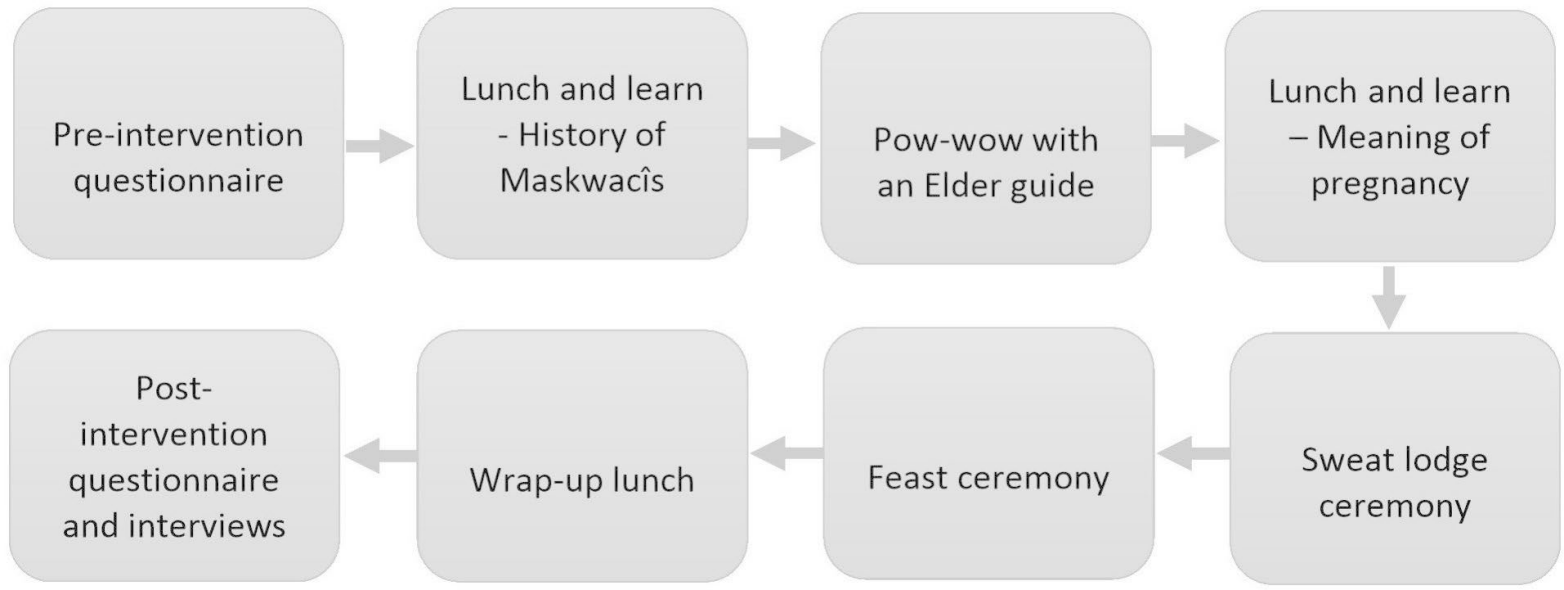
Study timeline

Cultural training can be conceptualized along a spectrum, from understanding the culture of others (cultural awareness) to understanding processes of cultural identity formation and one’s own positioning within this (cultural safety) [2]. It can include a wide variety of activities, ranging from passive to highly interactive learning experiences. We combined cultural awareness and experiential learning processes, and didactic and interactive learning activities. The activities were specifically designed to bring participants to the community, provide space for relationship building, and immerse them in positive cultural experiences. The intervention took place over three months, provided a set of community-driven experiential learning activities, and all perinatal staff at the PCN and MHS were invited to voluntarily participate (Fig. 1). As noted above, experiential learning can be described as ‘learning by doing’ and then reflecting on the experience and using the new knowledge gained through this process in future situations [22]. This type of learning is widely used within Nehiyaw culture including within ceremony, on the land, and through other cultural activities. The two organizations were selected because they provide most of the perinatal care in the Maskwacîs area.

The selected number of participants for the quantitative phase was based on the number of HCPs and staff employed at each clinic, and their availability to in participate in sessions and complete questionnaires. To be included, participants needed to be employed at MHS or the PCN, and specifically work with Indigenous maternal health clients. All other HCPs and staff were excluded. To participate in the qualitative phase, the HCPs and staff had to have completed the post-intervention questionnaires and indicated they were interested in being interviewed. All participants were health professionals or part of the clinic administration.

Intervention activities included attending two separate lunch and learn events facilitated by Elders and community members on the history of Maskwacîs and what pregnancy means to Nehiyawak people, attending a pow-wow with an Elder guide, experiencing a sweat lodge ceremony, participating in a feast ceremony, and a wrap-up lunch with Elders to answer participant questions. Not all activities focused specifically on perinatal care, though all were planned to give HCPs and staff a better understanding of the Nehiyaw culture and context. To our knowledge, this is the first time the PCN and MHS had partnered towards enhancing cultural safety.

### Quantitative data and analysis

Participants’ self-reported cultural knowledge and comfort levels in engaging with the community were assessed using two questionnaires, administered pre- and post-intervention. The validated Cultural Intelligence Scale (CQS) is a 20-item questionnaire that characterizes one’s cultural awareness according to four domains: metacognitive, cognitive, motivational, and behavioral[23]. The CQS has been validated across time, in several different populations, and in different geographic regions [24–26]. The four CQS domains are: Specifically, the four CQS domains are: metacognitive (4 statements), defined as the degree to which a person plans for, remains aware during, and reflects after multicultural interactions; cognitive (6 statements), defined as the level of a person’s understanding about how cultures are similar and how they are different; motivational, (5 statements), defined as the level of a person’s interest, persistence, and confidence to function in culturally diverse settings; and behavioural (5 statements), defined as the extent of a person’s flexibility and appropriate use of a broad repertoire of behaviours and skills during multicultural encounters. All items use a 7-point Likert scale, and values for each item are summed for a total score. There are no defined cut-points for the total score or within a given domain.

The Maskwacîs-Specific Cultural Scale (MSCS) is a 12-item questionnaire developed iteratively with the CAC as a part of the current study, and informed by scoping interviews with community members. The scoping interviews were transcribed and themes were identified. The themes were then crafted into a questionnaire and brought to the CAC for further refinement and discussion. The CAC had final say on the questions were used in the MSCS. The MSCS was designed to assess cultural awareness specific to Maskwacîs and contains questions related to self-reflection and self-awareness in providing culturally safe care; these questions also use a 7-point Likert scale and are summed for a total score. The MSCS was community-developed and piloted with several community members before use, however it was not validated for validity or reliability through statistical testing.

Initial data exploration indicated the data was not normally distributed. Descriptive statistics (mean; SD) were calculated for each item, and the main effects of time (pre vs. post), clinic (PCN vs. MHS), and interactions were assessed using Wilcoxon signed rank test (via Stata version 16.1). Two-sided p-values of 0.05 or lower was considered statistically significant. Discussions with the CAC indicated that participant responses to questionnaires would likely vary depending on whether they worked within the community or within surrounding areas. Thus, pre- and post-intervention scores were compared by location (PCN vs. MHS) as well as for both groups combined. Detailed demographic information was not collected in consideration of participants’ anonymity.

### Qualitative data and analysis

We used qualitative description to further understand the impact of the intervention. Participants self-identified as willing to be interviewed on the post-intervention questionnaire following completion of all the interactive sessions. They took part in one-to-one semi-structured interviews within four months post-intervention. Interviews were audio recorded and transcribed verbatim. Participants chose the setting and time for their interview (local coffee shop, private room in one of the clinics) and all interviews were led by the first author (GB). The interview guide was developed with the CAC and included questions about how participants work with Indigenous families that they care for, facilitators and challenges they experience in their work, their experiences with the intervention activities, the impact (positive or negative) they attributed to participating in the activities, and suggested changes to the activities that were offered. Thematic analysis (using ATLAS.ti software) and data generation were conducted concurrently by GB and RTO [27]. To code, the data transcripts were read and re-read separately, and persistent concepts and core patterns were highlighted and categorized. Highlighted sections were excised and grouped in categories. Categories were similarly read and re-read and subcategories were created. Emerging categories were compared together and in relation to the entire data set to identify common threads. Next, the CAC were presented with the initial findings and convened for a series of meetings aimed at refining categories, reducing redundancy, and ensuring accuracy and community appropriateness of the findings. The findings were considered complete when no additional insights emerged and when categories were well defined and refined by the CAC.

The CAC identified seven CBPR principles specific to Maskwacîs: trust, building strong relationships, mutual benefits, equitable partnership, shared knowledge, incorporating cultural teachings, and action-oriented and strengths-based research. Adhering to these principles ensured rigor in our study. Moreover, rigor was enhanced by providing participants with the opportunity to validate the findings. The overall findings were presented at individual meetings with qualitative participants from the PCN and MHS to get feedback and ensure their collective voices were accurately portrayed.

## Results

### Quantitative results

Of the approximately 30 eligible participants from both clinical settings, 20 completed pre-intervention questionnaires (PCN, n = 12; MHS, n = 8) and 17 completed post-intervention questionnaires (PCN, n = 8; MHS, n = 9). Participants were physicians (n=4), nurses (n=7) , other medical (n=4), or clerical/managerial staff (n=5). Attendance for the learning activities varied: history of Maskwacîs lunch and learn (n = 11); Cree pregnancy lunch and learn (n = 11); Samson Cree Nation pow-wow (n = 3); sweat lodge ceremony (n = 4); feast ceremony (n = 12); question and answer wrap-up lunch (n = 15).

Total summed CQS scores increased significantly after the intervention (p = 0.04), primarily driven by increases among participants from the PCN (p = 0.03) (Table 1). Specific increases pre- to post-intervention among the PCN-based participants were mainly within the metacognitive and cognitive domains, with significant increases in scores on consciousness of (p = 0.04), adjusting (p = 0.03), and checking the accuracy of cultural knowledge (p < 0.01; p = 0.03 for interaction). Scores on items related to knowledge around language (p < 0.01), marriage systems (p = 0.04), and non-verbal behaviors (p = 0.02) also increased significantly among PCN-based participants. Although it was not statistically significant, the knowledge of cultural and religious beliefs increased over time among PCN-based participants (p = 0.12) and did not change among the MHS group (p = 0.03 for interaction). We observed a significant interaction on an item from the motivation domain indicating that PCN-based participants were more confident socializing with cultures unfamiliar to them after the intervention (p = 0.03), whereas there was no change among MHS staff.

**Table 1:** CQS response scores from HCPs and staff from Maskwacîs (MHS) and Wetaskiwin (PCN) pre- and post-intervention. Values are means (SD).

Questionnaire Statement	Pre-MHS (n = 8) Mean (SD)	Post-MHS (n = 9) Mean (SD)	Pre-PCN (n = 12) Mean (SD)	Post-PCN (n = 8) Mean (SD)
Mcog1: I am conscious of the cultural knowledge I use when interacting with people with different cultural backgrounds.	6.25 (0.87)	6.22 (0.67)	5.16 (0.94)	5.88 (0.35)*
Mcog2: I adjust my culture knowledge as I interact with people from a culture that is unfamiliar to me.	6.38 (0.74)	6.22 (0.83)	5.33 (0.78)	6.125 (0.64)*
Mcog3: I am conscious of the cultural knowledge I apply to cross cultural interactions.	6.50 (0.53)	6.44 (0.53)	5.33 (0.89)	5.50 (0.76)
Mcog4: I check the accuracy of my cultural knowledge as I interact with people from different cultures.	6.63 (0.52)†	6.22 (0.83)†	4.83 (1.19)†	5.75 (0.98)†*
Cog1: I know the legal and economic systems of other cultures.	4.88 (1.25)	4.89 (1.45)	3.58 (1.44)	4.13 (1.55)
Cog2: I know the rules (e.g., vocabulary, grammar) of other languages.	3.38 (1.06)	4.22 (1.20)	2.83 (1.27)	4.63 (1.19)*
Cog3: I know the cultural values and religious beliefs of other cultures.	5.25 (1.04)†	4.89 (0.93)†	4.00 (1.13)†	5.13 (0.83)†
Cog4: I know the marriage systems of other cultures.	4.50 (1.20)	4.67 (1.00)	3.42 (1.44)	4.75 (1.12)*
Cog5: I know the arts and crafts of other cultures.	4.88 (1.55)	5.11 (1.17)	4.25 (0.97)	5.00 (0.76)
Cog6: I know the rules for expressing non-verbal behaviours in other cultures.	4.13 (1.13)	5.11 (1.17)‡	3.83 (1.11)	4.88 (0.35)*‡
Mot1: I enjoy interacting with people from different cultures.	6.75 (0.46)	6.89 (0.33)	6.67 (0.49)	6.75 (0.46)
Mot2: I am confident that I can socialize with cultures unfamiliar to me.	6.25 (0.71)†	5.89 (0.78)†	5.67 (0.89)†	6.50 (0.76)†
Mot3: I am sure I can deal with the stresses of adjusting to a culture that is new to me.	6.00 (1.07)	6.33 (0.71)	5.92 (0.79)	6.25 (0.46)
Mot4: I enjoy living in cultures unfamiliar to me.	5.38 (1.51)	5.89 (1.05)	5.50 (1.09)	5.38 (1.41)
Mot5: I am confident that I can get accustomed to the shopping conditions in a different culture.	5.13 (1.55)	5.56 (0.88)	6.00 (0.60)	5.63 (0.92)
Beh1: I change my verbal behaviour (e.g., accent, tone) when a cross cultural interaction requires it.	6.00 (1.07)	5.78 (1.20)	5.67 (0.78)	5.50 (0.93)
Beh2: I use pause and silence differently to suit different cross-cultural situations.	5.75 (1.04)	5.67 (0.50)	5.00 (1.41)	5.50 (0.93)
Beh3: I vary the rate I speak when a cross cultural situation requires it.	5.63 (0.92)	5.67 (0.50)	5.50 (0.90)	5.75 (0.71)
Beh4: I change my non-verbal behaviour when a cross-cultural situation requires it.	5.75 (1.04)	5.58 (0.79)	5.67 (0.50)	5.75 (0.89)
Beh5: I alter my facial expression when a cross-cultural interaction requires it.	5.13 (1.36)	5.67 (0.50)	5.25 (0.75)	5.75 (1.04)
CQS sum total	110.50 (14.11)	113.00 (10.72)‡	99.33 (9.41)	110.50 (10.07)*‡

* Significant difference (p < 0.05) between pre and post for PCN group

† Significant interaction effect (p < 0.05)

‡ Significant difference (p < 0.05) between pre and post for both PCN and MHS groups together

Total summed MSCS score increased significantly after the intervention (p = 0.01), again driven by increases among participants from the PCN (p < 0.01) (Table 2). Scores on five items improved for the PCN and MHS groups when combined: reflecting on increased awareness about Maskwacîs culture (p < 0.01), individual biases when interacting with women from the community (p = 0.02), understanding reasons for missed appointments (p = 0.04), support resources available within the community (p = 0.02), and the historical processes that influence health and culture (p < 0.01).

**Table 2:** MSCS scores from HCPs and staff from Maskwacîs (MHS) and Wetaskiwin (PCN) pre- and post-intervention. Values are means (SD).

Questionnaire Statement	Pre-MHS (n = 8) Mean (SD)	Post-MHS (n = 9) Mean (SD)	Pre-PCN (n = 12) Mean (SD)	Post-PCN (n = 8) Mean (SD)
I feel that I am aware about the culture of Maskwacîs.	5.50 (1.31)	6.11 (0.33)‡	3.75 (1.42)	5.25 (0.71)*‡
I feel safe and welcome when experiencing the community of Maskwacîs.	6.25 (1.17)	6.44 (0.73)	4.50 (1.68)	5.50 (1.60)
I feel that Maskwacîs culture is dynamic and may vary from community to community and family to family.	6.88 (0.35)	6.67 (0.50)	6.08 (1.08)	6.63 (0.52)
I feel that I can communicate well with individuals from Maskwacîs.	6.38 (1.06)	6.67 (0.50)	5.67 (0.65)	6.13 (0.64)
I feel that I have a good understanding of the reasons some women from Maskwacîs may miss appointments and/or not come in for care.	6.38 (0.74)	6.22 (1.99)‡	5.92 (0.79)	6.50 (0.53)‡
I feel that I am able to adapt easily when interacting with women from Maskwacîs when needed.	6.38 (0.74)	6.56 (0.53)	5.83 (0.83)	6.50 (0.53)
I feel that I have an appropriate amount knowledge about the resources available to support women and their partners in different communities in Maskwacîs.	5.88 (1.25)	6.44 (0.53)‡	4.25 (1.29)	5.38 (1.69)‡
I feel that I am aware of the historical processes that influence health and culture within Maskwacîs today.	5.38 (1.19)	6.33 (0.50)‡§	4.25 (1.22)	5.75 (0.89)*‡
I feel that I am aware of my own biases when interacting with pregnant women from Maskwacîs.	5.63 (1.06)	6.44 (0.53)	4.83 (1.12)	5.38 (0.92)
I am aware of my body language when interacting with individuals from Maskwacîs.	6.25 (1.04)	6.33 (0.71)‡	5.25 (0.87)	5.75 (0.46)‡
I feel that relationship building and maintenance plays a key role in enhancing cultural security.	6.88 (0.35)	6.78 (0.44)	6.67 (0.65)	6.75 (0.46)
I feel that self-reflection is important in interacting with individuals from Maskwacîs.	6.88 (0.35)	6.89 (0.33)	6.08 (1.00)	6.38 (0.74)
MSCS sum	74.63 (7.89)	77.89 (5.49)‡	63.08 (6.22)	71.88 (5.91)*‡

* Significant difference (p < 0.05) between pre and post for PCN group

‡ Significant difference (p < 0.05) between pre and post for both PCN and MHS groups together

§ Significant difference (p < 0.05) between pre and post for MHS group

### Qualitative results

Nine participants were interviewed (PCN, n = 4; MHS, n = 5) after completion of the learning opportunities. Participants were physicians (n = 3), nurses (n = 3), other medical (n = 1) (collectively called HCPs), or clerical/managerial staff (n = 2) (staff). They shared compelling reflections on their experiences during the intervention activities. The most cited theme offered by participants was 1) the personal impact of participation, and the next most frequently cited theme was how participation positively influenced their ability to provide culturally informed care. Participants felt that overall, the intervention created a space for them to experience the community in ways they had not done before. By providing an opportunity for HCPs and staff to make connections within the community, the intervention was able to address fears or uneasiness the participants may have felt beforehand both personally as well as within their role as a clinical care provider or staff member.

### Personal impact

Many of the participants gave credit to and appreciated the firsthand experiential nature of the activities, which resulted in personal, meaningful, moving, and even spiritual experiences. Participants noted that the intervention activities provided a time and space for HCPs and staff to build relationships within the community and connect with community members, other HCPs and staff, and Nehiyaw culture. The ceremonial intervention activities were particularly transformative. One participant shared: “The sweat lodge was an amazingly eye-opening experience for me, emotionally and spiritually on a very personal level … I think it was sort of the gateway for me to be comfortable to ask about culture, and ask about, you know, ceremony and tradition.” (Health Care Provider).

Moreover, the intervention activities allowed participants to see many positives and strengths within the community, rather than a largely inaccurate portrayal perpetuated through negative stereotypes, media, or crisis encounters in clinical care.

Participants spoke at length about how the activities increased their overall cultural knowledge as it relates to Maskwacîs. Specifically, intervention activities provided an opportunity for participants to build meaningful and personal connections with community members and to learn about family kinships, cultural practices and norms, traditional approaches to well-being, individual ceremonies and their meanings, as well as other social aspects of the community. For example, one participant explained that they “learned more of the sensitive side of things, you know, how important family is and not just your blood family, but like extended family. That someone will help you if they can.” (Health Care Provider) Participants also expressed a collective sense of enjoyment and a desire to participate in ongoing and future activities of a similar nature.

### Impact on clinical Care

After experiencing the intervention activities, participants felt emboldened and better equipped to provide more culturally informed care to their patients. Providing a positive opportunity for HCPs and staff to experience Nehiyaw culture firsthand was discussed as a key take away for participants. For instance, participants spoke of taking more opportunities to meaningfully engage on a personal level and build relationships with patients. They used their experiences with the intervention activities as conversation starters or icebreakers with patients, and found that talking about the community and its culture helped create a safer space for meaningful interaction. Participants felt more comfortable discussing and asking patients about their own culture, which provided a point of mutual understanding to gain and build trust. One participant expressed: “There’s that sense of connecting which I think is really, really important. So, if one my clients see me out there (in Maskwacîs) they will know that I am not stand-offish toward their culture. I am willing to be a part of it when it is appropriate.” (Health Care Provider).

Some participants noted that their experience directly changed their professional practice in that they now encourage patients to consider incorporating ceremony and engaging with Elders into their treatment plan. One participant expressed having a new and expanded view of health and that this impacts how they provide clinical care: “I do think that ceremony has an important role in peoples’ health and now that I understand the ceremony it is much easier to make it a recommendation rather than an option.” (Health Care Provider).

Additionally, enhanced cultural and social awareness and insight into the day-to-day lives of community members allowed participants to feel more empathy and understanding. For example, participants expressed a desire for more flexibility and leniency in the clinical setting around common transportation issues and missed appointments of patients.

## Discussion

Our findings show that a series of Indigenous-led and culturally based experiential learning opportunities was effective in enhancing aspects of perceived cultural awareness for local perinatal HCPs and staff, and that participants felt this improved their preparedness to provide culturally informed care. Through a meaningful community-led approach, the activities used in this study provided HCPs and staff with an opportunity to experience the community of Maskwacîs in ways that many had not done before. Often, CBPR is conducted predominately by outsiders and therefore may not allow for a nuanced approach to research that is required to navigate social, political, cultural and familial dynamics within Indigenous communities. The genuine partnerships developed through our collaboration allowed for the CAC to be involved in all aspects of the study, and provided the platform for a community member (GB) to facilitate and lead the study. As a result, our community partners feel our research was novel, impactful, and truly reflective of the wants and needs of the community.

After the intervention activities, participants perceived an increase in cultural and community knowledge and higher comfort levels in engaging with the community, with significant positive gains in overall mean scores on both the CQS and MSCS questionnaires. The questionnaires also showed that HCPs, specifically the ones who did not work in community, were more motivated and comfortable in social interactions with community members, whereas those that worked in community already felt comfortable in those settings. The development and implementation of the nation-based questionnaire in the form of the MSCS allowed the community to include elements that are important to them, and to quantify changes in perinatal health care in their community as a result of research.

Qualitative findings revealed personally meaningful, impactful, and enjoyable experiences from participation in the intervention activities that enhanced their overall sense of cultural knowledge and laid a foundation for more culturally informed patient interactions. HCPs and staff described their intervention experiences, reflected on how much they had learned about the distinctions of the community and culture, such as kinship and how Nehiyawak people view family. As a result, they felt more prepared for future clinical interactions with families from the community. They also felt that they could visit the community more in the future after having experienced some cultural events.

There is a need for improved health system interactions for Indigenous patients; access to culturally informed and equitable health care has the potential to address Indigenous health inequities [28]. A review of Indigenous cultural training programs in Australia suggests training that focuses solely on cultural awareness to improve health services is insufficient [29]. Similarly, our previous qualitative research and the work of others [8, 30, 31] suggests that passive cultural awareness activities alone rarely result in effective culturally informed care, and that incorporating shared experiential learning activities that allow for self-reflection is needed. Few studies assess the impact of cultural training activities that go beyond the acquisition of cultural knowledge. As far as we are aware, ours is the first to examine the impact of experiential learning activities among perinatal HCPs and staff. Similarly, a qualitative study in which nursing students took part in several two-week intensive learning activities in the circumpolar north also showed a positive impact on student perspectives of the social determinants of health and well-being relevant to the local context [32].

Evidence supporting the impact of experiential learning on cultural awareness and safety is sparse, particularly among practicing HCPs and clinic staff. To date, cultural training initiatives have taken a mostly pan-Indigenous approach, where Indigenous peoples, cultures, and histories are generalized for simplicity and convenience [33]. However, Indigenous peoples in Canada encompass more than 650 recognized individual Nations with diverse cultures, languages, histories, and sociopolitical and socioeconomic factors [34]. Using a decolonizing approach, our intervention was a community-based effort reflecting the local context.

### Future directions

The experiential learning approach utilized in this study could also be used with other clinics, hospitals, and organizations that provide services for Indigenous families. Although qualitative findings provided some evidence that our intervention led to more culturally informed approaches to care, more research is needed on HCPs behavioral outcomes to form a stronger base of evidence, as well as impact assessments on patient experiences and their health outcomes. While our ongoing efforts currently focus on sustained involvement in community-based experiences and challenges presented by the ongoing COVID-19 pandemic, there could also be opportunities to better understand which experiential learning activities were most meaningful for involving HCPs and staff in the community or shifting their perceptions. The development and use of the MSCS was novel and allowed the community to develop and implement a tool that captures nation-specific important outcomes using a questionnaire tool. This approach can be attractive to other communities as a way to enhance the meaning and applicability of research, and address the communities wants and needs in a more direct way. Further work in nation-based research instruments, such as the Maskwacîs-Specific Cultural Scale (MSCS), is needed.

### Limitations

Most cultural training initiatives, including the one described for this study, heavily rely on self-reported measures, and so participants may have overestimated their own cultural awareness [35]. Since our study lacked a control group, there was no way to test for the impact of social desirability on responses to the post-intervention questionnaire. Additionally, participation was voluntary, and our study may have attracted a small group of HCPs and staff who were keen to enhance their delivery of culturally informed care. The MSCS has not been evaluated for validity or reliability by traditional western statistical methods, although it does represent the important perspectives of the community. While the overall approach to developing this instrument could be considered, we caution against using the same exact questionnaire in other Indigenous communities.

Not all participants took part in each of the intervention activities, limiting our ability to assess the potential impact on perceived cultural awareness. Also, three participants who completed the initial questionnaire did not complete the final one. Although this is a relatively small loss to follow-up, it should be considered when interpreting the data collected. Finally, each community is distinct, so transferring our findings to other Indigenous populations must be approached with caution. The small sample size and attrition are limitations, but provide a valuable and optimistic foundation for future collaboration, evaluation, and learning.

## Conclusion

Our study sought to design and implement an Indigenous-led intervention, to measure its effectiveness, and to capture changes in the preparedness and willingness of HCPs and staff to provide culturally informed care. The findings have valuable implications for those involved in the health care of Indigenous women and families. The findings show that using a decolonizing approach with experiential learning opportunities that are community-derived and culturally specific can increase components of self-reported preparedness to provide culturally informed care. This study provides evidence for fostering relationships between HCPs, health care systems, and Indigenous communities to develop and implement unique and meaningful learning opportunities. These efforts go beyond those typically used to improve cultural awareness (i.e., by passive means), but rather, center experiences and learning around the local knowledge of the community, allowing the community to be an active partner in the development and implementation of an culturally intervention that aims to enhance care.

## Data Availability

This study adheres to the principles of Ownership, Control, Access, and Privacy (OCAP™) and will not make the data publicly available.
